# A Computational Protocol for Vibrational Circular Dichroism Spectra of Cyclic Oligopeptides

**DOI:** 10.1021/acs.jpca.2c02953

**Published:** 2022-08-05

**Authors:** Karolina Di Remigio Eikås, Maarten T. P. Beerepoot, Kenneth Ruud

**Affiliations:** † Hylleraas Centre for Quantum Molecular Sciences, Department of Chemistry, UiT The Arctic University of Norway, 9037 Tromsø, Norway; ‡ Norwegian Defence Research Establishment, P.O. Box 25, 2027 Kjeller, Norway

## Abstract

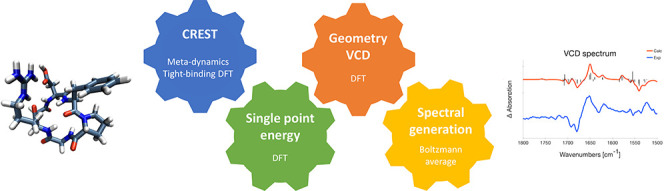

Cyclic peptides are a promising class of compounds for next-generation antibiotics as they may provide new ways of limiting antibiotic resistance development. Although their cyclic structure will introduce some rigidity, their conformational space is large and they usually have multiple chiral centers that give rise to a wide range of possible stereoisomers. Chiroptical spectroscopies such as vibrational circular dichroism (VCD) are used to assign stereochemistry and discriminate enantiomers of chiral molecules, often in combination with electronic structure methods. The reliable determination of the absolute configuration of cyclic peptides will require robust computational methods than can identify all significant conformers and their relative population and reliably assign their stereochemistry from their chiroptical spectra by comparison with *ab initio* calculated spectra. We here present a computational protocol for the accurate calculation of the VCD spectra of a series of flexible cyclic oligopeptides. The protocol builds on the Conformer-Rotamer Ensemble Sampling Tool (CREST) developed by Grimme and co-workers (Phys. Chem. Chem. Phys.2020, 22, 7169−71923207307510.1039/c9cp06869d and J. Chem. Theory. Comput.2019, 15, 2847–28623094302510.1021/acs.jctc.9b00143) in combination with postoptimizations using B3LYP and moderately sized basis sets. Our recommended computational protocol for the computation of VCD spectra of cyclic oligopeptides consists of three steps: (1) conformational sampling with CREST and tight-binding density functional theory (xTB); (2) energy ranking based on single-point energy calculations as well as geometry optimization and VCD calculations of conformers that are within 2.5 kcal/mol of the most stable conformer using B3LYP/6-31+G*/CPCM; and (3) VCD spectra generation based on Boltzmann weighting with Gibbs free energies. Our protocol provides a feasible basis for generating VCD spectra also for larger cyclic peptides of biological/pharmaceutical interest and can thus be used to investigate promising compounds for next-generation antibiotics.

## Introduction

Chirality is a key property of many biological systems. The two enantiomers of a chiral molecule may have very different biological functions^1^ with the extreme case being one enantiomer with a biological effect as a drug and the mirror image with an adverse effect when administered to a patient. This makes it important to be able to identify the stereochemistry of chiral molecules and to devise stereoselective synthetic pathways that can ensure that a particular enantiomer is synthesized. As almost all physical properties of two enantiomers are the same, the identification and separation of different enantiomers is challenging. The observation that enantiomers of chiral molecules interact differently with circularly polarized light has been used to develop a wide range of different chiroptical spectroscopies^2−4^ in which the differential absorption or scattering of right- and left-circularly polarized light gives rise to a different sign for the two enantiomers. However, there is no way to *a priori* connect the sign of the differential absorption of the components of the circularly polarized light to the absolute stereochemistry of the molecule, making it important to compare the experimental spectra with spectra calculated using electronic structure methods.

Circularly polarized light can be generated in a wide frequency range. Historically, chiroptical spectroscopies in the optical and near-UV region, first measured by Cotton in 1895,^5,6^ have been the most important methods for the discrimination of enantiomers. Indeed, optical rotation remains today a key quantity to determine, for instance, enantiomeric excess and to verify the stereochemistry of a chiral molecule. In order to obtain more insight into the electronic structure of chiral molecules as well as to determine the amount of α-helical content in proteins, electronic circular dichroism (CD) is commonly used.^7−9^ A significant limitation of both optical rotation and CD for the determination of the chirality of small molecules is the limited number of electronic excited states available in the wavelengths accessible to experimental investigation in the UV/vis region. This is particularly critical in the case of organic molecules, which often are colorless due to the fact that there are no low-lying excited states absorbing in the visible region, leaving only a very narrow window in the near-UV region accessible to modern detectors. These challenges are further accentuated when multiple chiral centers are present in the molecule. Furthermore, the description of electronic excited states using for instance density-functional theory (DFT) is fraught with challenges.^10,11^ In contrast, methods that treat electron correlation explicitly are faced with challenges arising from the size of the molecules normally involved and the lack of symmetry.^12−16^

In contrast, the infrared region is a rich source of information for structural characterization and identification of chiral molecules. Even small molecules have a large number of vibrational modes that can be probed with spectroscopies using the infared region of the electromagnetic spectrum, providing a much richer source of experimental data compared to electronic spectroscopies. Many of these vibrational modes can be associated with specific, local regions of the molecule. From a computational point of view, vibrational chiroptical spectroscopy also benefits from the fact that all relevant quantities can be calculated from a knowledge of the electronic ground state only,^17,18^ which in general can be determined more accurately than electronic excited states.

Since their first observations in the early 70s,^19−21^ vibrational chiroptical spectroscopies have evolved into some of the most important techniques by which the absolute stereochemistry of a molecule can be determined in a combined experimental/theoretical approach.^22,23^ The two most important vibrational chiroptical techniques are vibrational circular dichroism (VCD)^24−27^ in which one measures the differential absorption of left- and right-circularly polarized light, and Raman optical activity (ROA), in which one measures the differential scattering of right- and left-circularly polarized light.^2,28^ VCD and ROA are the chiroptical analogues of infrared absorption spectroscopy and Raman scattering, respectively. Of these, VCD is the most common approach, to a large extent due to the availability of several different commercial instruments and easier operation. VCD has found a wide range of applications, including structural characterization of small molecules,^29−34^ understanding the secondary structure of peptides and proteins^35−42^ as well as for the understanding of molecular behavior and interactions in solutions,^43−51^ and recently also for understanding interactions in larger molecular systems such as fibrils.^52−54^ The sensitivity of the approach to even small changes in molecular structure is one of the strengths of the technique. At the same time, this sensitivity puts severe demands on the robustness of the computational model in general, and the description of molecular interactions and solvent effects in particular.

From a computational point of view, a challenge in the study of larger chiral systems is conformational flexibility due to the strong dependence of the chiroptical response on the three-dimensional structure of a molecule.^55−57^ Indeed, the sign of the optical rotation as well as VCD absorption bands may change for different conformations of a molecule. The strong sensitivity on the molecular conformation also means that the calculated chiroptical response will be very sensitive to conformational sampling, both in terms of identifying all conformations present in solution, as well as the quality of the energetics used to determine the Boltzmann population of individual conformations. The complexity of this problem increases with increasing molecular size and the number of chiral centers in the molecule. The conformational sampling is thus crucial in order to reliably assign the stereochemistry of these molecules and is as such an integral part of any computation of chiroptical spectra of flexible molecules. The majority of studies in the literature use *ad hoc* schemes for conformational sampling, either based on chemical intuition or on sampling from molecular dynamics (MD) simulations.^58^ In the latter case, it is important that the conformational space is sufficiently well sampled.^59^ The conformational challenge gets further complicated when the molecule of interest also can form stable structures with solvent molecules, for instance, through hydrogen bonding. An early study of these effects was presented by Hopmann et al.,^59^ discussing, for instance, the use of either the enthalpy or the free energy for determining Boltzmann weights as well as the challenges of comparing complexes with different number of solute–solvent bonds. Despite a large number of studies of chiroptical responses of conformationally flexible molecules in recent years,^58−63^ there is a need for a more systematic approach to the sampling of conformations for flexible molecules to provide a robust and reliable approach for calculating vibrational chiroptical spectra of chiral molecules with multiple chiral centers.

At the same time, the conformational challenge is not unique to chiroptical spectroscopy. A number of strategies for sampling the conformation space of small organic molecules have been presented.^64−68^ A particular promising approach was recently presented by Grimme and co-workers,^69−71^ originally developed for the study of spectroscopic properties of flexible molecules with the first application being the calculation of the nuclear magnetic resonance (NMR) spectrum of the cyclic ionophore nonactin in addition to a few other (in)organic and transition-metal complexes. The approach has since been further refined and applied to the calculation of different spectroscopies, including VCD.^71−75^ As such, this approach appears as a promising starting point for a robust and reliable protocol for calculating VCD spectra of conformationally flexible molecules with multiple stereocenters.

**Figure 1:**
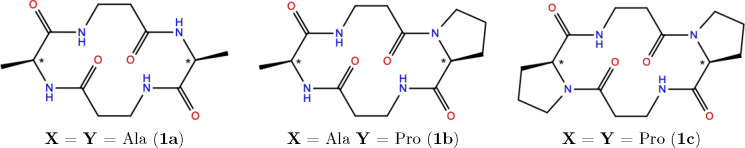
Structure of cyclo(**X**-β-Ala-**Y**-β-Ala) with the chiral centers indicated by an asterisk.

**Figure 2:**
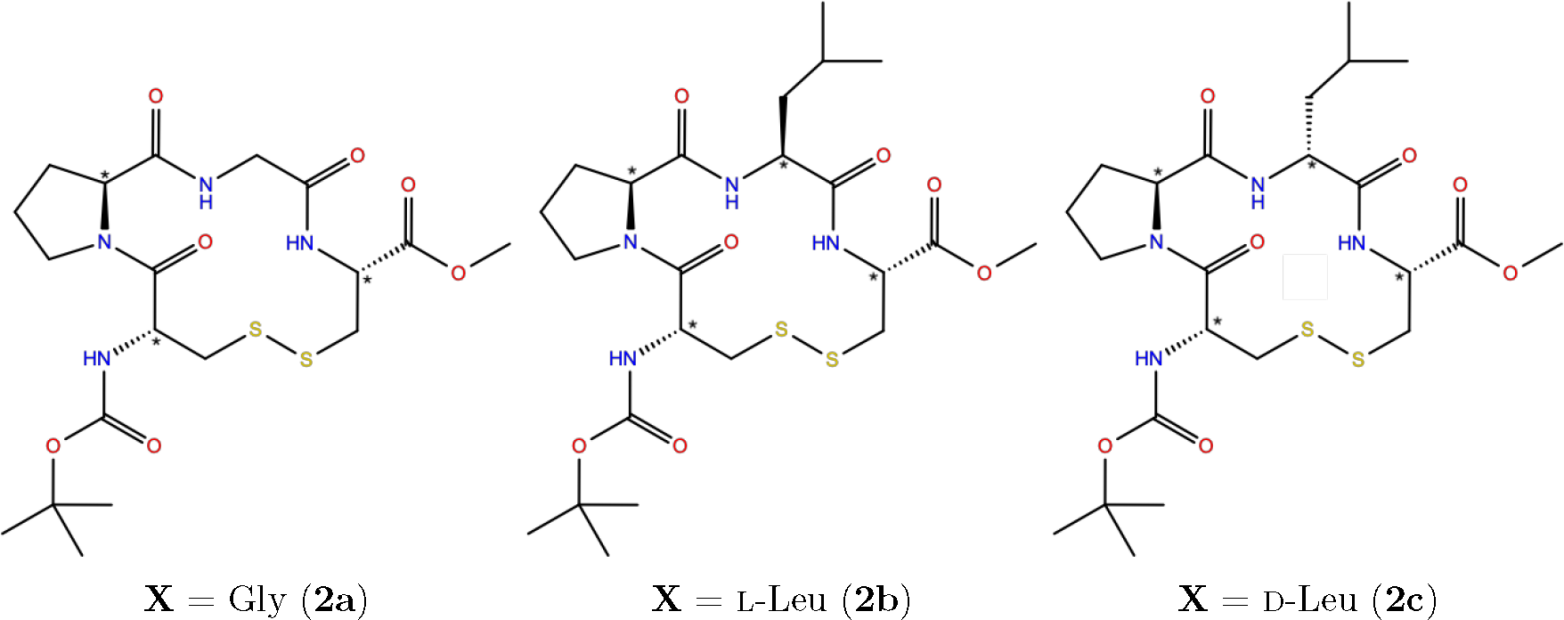
Structure of cyclo(Boc-Cys-Pro-**X**-Cys-OMe) with the chiral centers indicated by an asterisk.

**Figure 3:**
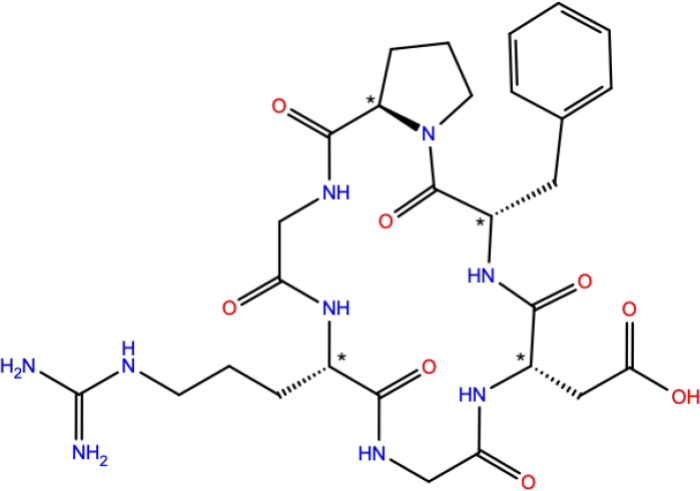
Structure of cyclo(Phe-d-Pro-Gly-Arg-Gly-Asp) (**3**) with the chiral centers indicated by an asterisk.

Our primary targets are cyclic oligopeptides in the so-called middle space,^76^ as these systems hold promise for novel actions as antibiotics. It is important that the computational protocol is tested on well-known systems that share similarities with the compounds of interest, while at the same time being simple enough to allow the protocol to be suitably optimized and its range of applicability assessed. For this purpose we have selected three classes of cyclic oligopeptides previously reported in the literature.^77−79^ The first and simplest system is a series of three tetrapeptides of the form cyclo(**X**-β-Ala-**Y**-β-Ala) with **X** and **Y** both being either proline or alanine (see Figure 1). The second class is another series of three tetrapeptides of the form cyclo(Boc-Cys-Pro-**X**-Cys-OMe), where **X** can be glycine, l-leucine, or d-leucine, respectively (Figure 2). Finally, we apply the protocol to the hexapeptide cyclo(Phe-d-Pro-Gly-Arg-Gly-Asp) (Figure 3).

The rest of the paper is organized as follows: We first describe the details of our computational protocol, including the conformational sampling, the selection of conformers, their energy minimization and the calculation of VCD spectra. We then turn our attention to the optimization and use of the computational protocol for our reference systems, before we end by giving some concluding remarks and an outlook.

## Computational Details

**Figure 4:**
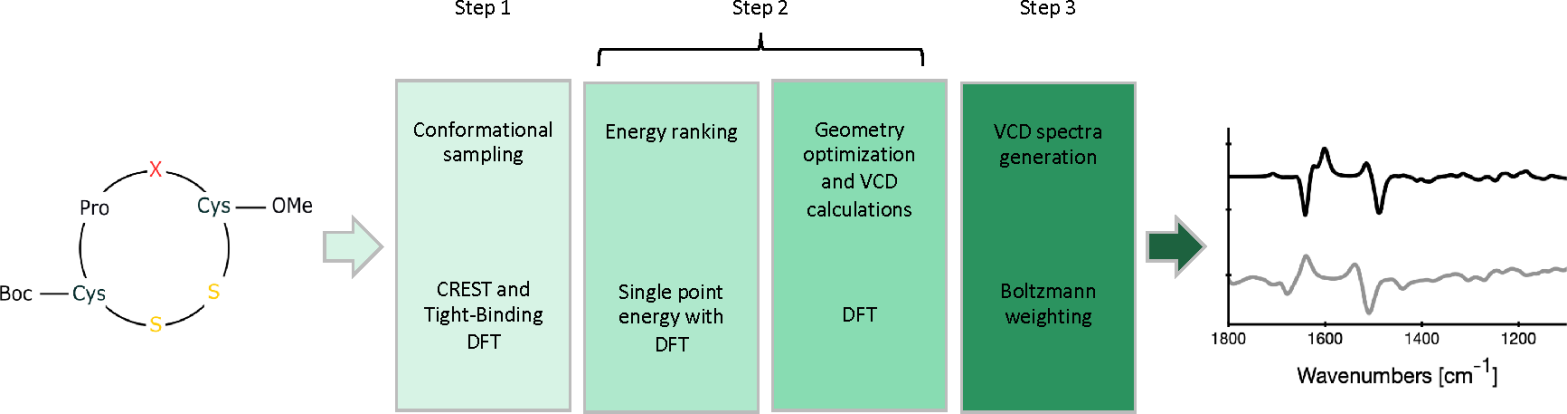
Overview of the protocol presented in this work.

Our computational protocol for the calculation of VCD spectra of conformationally flexible molecules such as the cyclic oligopeptides that are our primary targets, consists of three steps that are summarized in Figure 4: (1) sampling of the conformational space, (2) selection of the conformers that can be expected to be important for the VCD spectrum, structural optimization of these conformers and calculation of VCD spectra, and finally (3) the generation of the Boltzmann-averaged VCD spectrum that can be compared to the experimentally observed spectrum.

### Step 1: Conformational Sampling

We start by considering the selection of relevant conformers for which we used the Conformer Rotamer Ensemble Sampling Tool (CREST), version 2.8.^69−71^ In our calculations on molecules **1a**–**c** (Figure 1), **2a**–**c** (Figure 2), and **3** (Figure 3), we used the iMDT-GC workflow^70,71^ and the default settings with the exception of the simulation length of **1b**. The iMDT-GC workflow consists of three steps: an extensive meta-dynamic sampling (MTD) with different bias potentials, a molecular dynamics (MD) sampling around the lowest-energy conformers, and a genetic Z-matrix crossing (GC).^70,71,80^ The GC compares each pair of conformers *i* and *j* and adds the structural differences (*R*_*i*_ – *R*_*j*_) to a reference structure to generate a new conformer (*R*_new_ = *R*_ref_ + (*R*_*i*_ – *R*_*j*_)). The reference structure, *R*_ref_, is usually the conformer lowest in energy.

The following describes the default CREST settings with the iMDT-GC workflow: On the basis of a flexibility measure for the molecule, CREST determines the length of the MTD simulation, which typically is 0.3–0.4 ps multiplied by the number of atoms in the molecule. The resulting simulation length for each molecule in this work is given in Table 1. A time step of 5.0 fs was used, coordinates were sampled every 100 fs, and a new reference structure for the bias potential was chosen every 1.0 ps.^80^ The bias potential used for the MTDs combines 3 different prefactors (*k*), the number of atoms (*N*) times 0.00300, 0.0015, and 0.000075, and 4 exponents (α), 1.300, 0.780, 0.468 and 0.281, resulting in 12 different bias potentials and thus 12 MTD sequences. In addition, two MTD sequences with extreme bias potentials (*k* = *N* × 0.0010, α = 0.1 and *k* = *N* × 0.0005, α = 0.8) were performed. For each snapshot in the simulations, a loose geometry optimization with the tight-binding method GFN-xTB^81,82^ was performed. If this resulted in a conformer lower in energy than the input geometry, the MTD procedure was restarted with the geometry of this conformer. This was done at least once and no more than five times. For the four conformers lowest in energy, MD simulations were performed at 400 and 500 K, respectively. Finally, geometry optimizations using GFN-xTB with tighter convergence thresholds were performed for the conformers obtained from both the MTD and MD simulations. All structures thus obtained were compared and duplicates removed. CREST identifies duplicates based on three criteria: the total energy, the root-mean-square-deviation of atomic Cartesian coordinates, and the difference in the rotational constants. The final ensemble of unique conformers was then generated by performing a geometry optimization with very tight convergence criteria, again using GFN-xTB.

**Table 1:** Computational Settings for the Conformer Rotamer Ensemble Sampling Tool (CREST) for Each of the Molecules Studied in This Work

molecule		MTD time (ps)	MD time (ps)
**1a**	**X** = **Y** = Ala	13.0	6.5
**1b**	**X** = Ala, **Y** = Pro	90.0^a^	45.0^a^
**1c**	**X** = **Y** = Pro	11.0	5.5
**2a**	**X** = Gly	21.0	10.5
**2b**	**X** = l-Leu	28.0	14.0
**2c**	**X** = d-Leu	28.0	14.0
**3**		36.0	18.0

a Because of difficulties with finding conformers, the simulation time was set manually.

For molecule **1b**, the simulation length was manually set. For **1a** and **1c**, the conformers sampled with the simulation time determined by CREST resulted in VCD spectra in agreement with experiment and a conformer ensemble including the conformer determined most important by Vass et al.^77^ For **1b**, on the other hand, the simulated spectrum was not in agreement with experiment. CREST had difficulties finding the relevant conformers and the conformer determined as most important by Vass et al. was not in the ensemble. By increasing the simulation time from 12.0 ps suggested by CREST to 90.0 ps, the conformer ensemble included the relevant conformers.

In CREST 2.8, the solvent is described by a Generalized Born model where a solvent-accessible surface is used. The experimental data^77−79^ for **1b**–**c** and **2a**–**c** were recorded in deuterated acetonitrile (ACN-*d*_3_) and ACN (ϵ = 36) was used in the calculation. **1a** was measured in deuterated trifluoroethanol (TFE-*d*_2_) while **3** was measured in normal trifluoroethanol (TFE). Since TFE (ϵ = 27) is not available in CREST, we chose the available solvent with the most similar dielectric constant, acetone (ϵ = 20).

### Step 2: Energy Ranking and VCD Calculations

Having in step 1 identified a possibly complete set of unique conformers, we next turn to the calculation of VCD spectra for the energetically relevant conformers. In selecting the relevant conformers, we perform single-point energy calculations at the DFT level of theory, following the recommendation by Grimme.^70^ Single-point energies, geometry optimizations and VCD calculations were performed using DFT in Gaussian 16 (Rev. B.01).^83^ Building on previous studies of VCD calculations at the DFT level of theory,^29,84−87^ the B3LYP functional^26,88,89^ has been used for these calculations in combination with the 6-31+G*^90,91^ basis set. To evaluate the effect of the size of the basis set on the resulting VCD spectra for cyclic oligopeptides, single-point energies, geometry optimization, and VCD calculations were also performed using B3LYP with the 6-31+G, 6-31+G**, 6-31G*, 6-31++G*, and 6-311+G* basis sets for molecules **1a** and **2a**. These results can be found in the Supporting Information. In addition, Grimmes empirical dispersion correction D3 with Becke-Johnson damping^92^ was tested for molecules **1a** and **2a** (B3LYP-D3). Solvent effects were included using the conductor-like polarizable continuum model (CPCM).^93,94^

Geometry optimization and VCD calculations were performed on conformers that were at most 2.5 kcal/mol higher in energy than the most stable conformer after the single-point calculations. Both larger and smaller energy gaps where tested (*vide infra*). After geometry optimization with DFT, many conformers determined to be unique by CREST end up in the same minimum. Duplicates were removed with a script^95^ using the same set of criteria as used in CREST.

### Step 3: VCD Spectra Generation

Boltzmann weights for all unique conformers identified after the DFT geometry optimization were used to generate VCD spectra in the DrawSpectrum program.^96^ Gibbs free energies for the conformers were used to calculate the Boltzmann weights if not otherwise specified.

When comparing calculated spectra only, frequency scaling factors from Merrick, Moran, and Radom were used.^97^ When comparing calculated and experimental spectra, the frequencies were scaled such that the combined IR and VCD overlap estimates (eq 1) were maximized. A more detailed discussion of scaling factors when calculated spectra are compared with experimental spectra is found in the Supporting Information (SI). The overlap estimate between two spectra *a* and *b* was calculated with DrawSpectrum as^86,98−100^
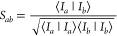
1where *I*_*a*_ is the spectral intensity at a given wavenumber for spectrum *a*. *S*_*ab*_ ranges from −1 to 1, where 1 indicates identical spectra and −1 perfect mirror-image spectra. The frequency regions used were 1800–1500 cm^–1^ for molecules **1a**–**1c** and **3** and 1800–1100 cm^–1^ for molecules **2a**–**c**.

The spectral line shape was simulated with a Lorentzian function with a full width at half-maximum of 10 cm^–1^ for molecules **1a**–**c**^77^ and **3**,^79^ and 16 cm^–1^ for molecules **2a**–**c**.^78^ The intensities in the calculated spectra are scaled only when comparing to experimental spectra, and in these cases the intensities are scaled such that the maximum intensity of the strongest absorption band matches the intensity of the corresponding band in the experimental spectrum.

## Results and Discussion

We now turn our attention to the optimization of the protocol by testing the effect of adding dispersion corrections, the selection of significant conformers, and an analysis of the relevant conformers. We then use the optimized protocol to predict IR and VCD spectra and compare to the experimental spectra of the selected molecules.

### Dispersion Corrections

**Figure 5:**
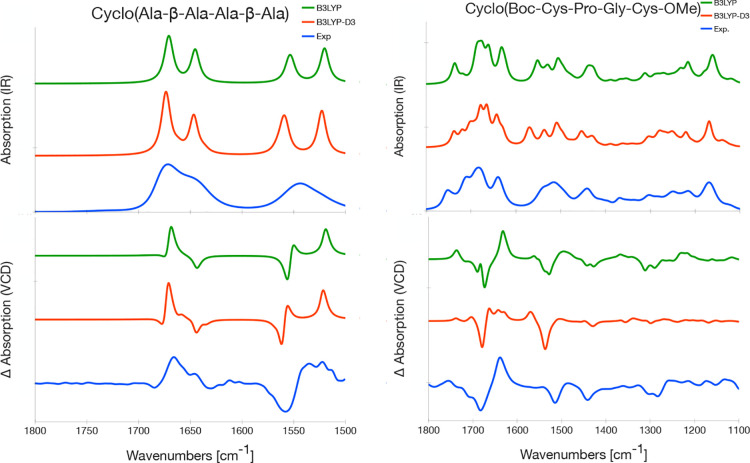
IR and VCD spectra of **1a** in TFE-*d*_2_ (left) and **2a** in ACN-*d*_3_ (right) with B3LYP/6-31+G*/CPCM and B3LYP-D3/6-31+G*/CPCM, compared with experiment. The frequencies were scaled with a factor of 0.975 for B3LYP-D3 on **2a** and 0.980 for B3LYP on **2a** as well as for all calculations on **1a**. The experimental spectrum of **1a** is measured by Vass et al.^77^ while the one of **2a** by Merten et al.^78^

**Figure 6:**
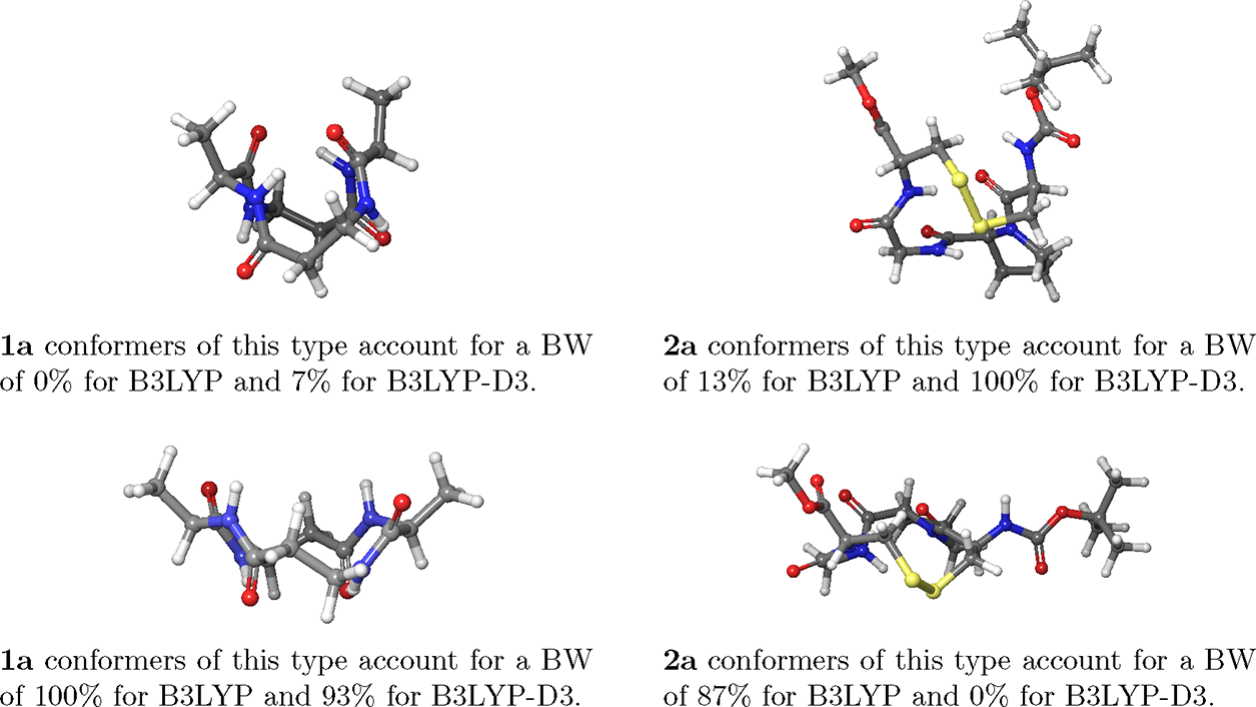
Computed geometries of **1a** (left) and **2a** (right) with and without dispersion interactions. The conformers can be grouped in two different types: the U-type (top row, with dispersion interactions) and flat type (bottom row, without dispersion interactions). The Boltzmann weights given in the figure are the sum of the conformers belonging to that group.

Dispersion corrections are automatically included in CREST calculations (step 1) through the D4 dispersion model.^101−103^ We here test the effect of including dispersion corrections also in steps 2 and 3, that is, single point energies, geometries, and VCD properties calculated with DFT. The effect of adding dispersion corrections has been tested on molecules **1a** and **2a** and the results are shown in Figure 5 (IR and VCD spectra), Figure 6 (computed geometries) and Table 2 (overlap estimates *S*).

**Table 2:** Overlap Estimate *S* between Calculated and Experimental Spectra with B3LYP and B3LYP-D3^a^

	molecule **1a**		molecule **2a**	
	IR	VCD	IR	VCD
B3LYP	0.86	0.68	0.93	0.70
B3LYP-D3	0.86	0.63	0.93	0.46

a *S* is calculated over the frequency range 1800–1500 cm^–1^ for **1a** and 1800–1100 cm^–1^ for **2a**, using the experimental spectrum as the reference. The frequencies were scaled with a factor of 0.975 for B3LYP-D3 on **2a** and 0.980 for B3LYP on **2a** as well as for all calculations on **1a**.

The IR spectra with and without dispersion corrections are similar, both qualitatively (Figure 5, top) and quantitatively (Table 2). Indeed, overlap estimates between calculated and experimental spectra are the same (0.86 for **1a** and 0.93 for **2a**) with and without dispersion corrections (Table 2). On the other hand, VCD is a much more sensitive technique. The most striking difference is the peak around 1630 cm^–1^ for **2a**, which is only reproduced by the spectrum without dispersion corrections (Figure 5). Accordingly, the overlap estimate for the calculated VCD spectrum is higher for B3LYP (0.70) than for B3LYP-D3 (0.46, Table 2). The effect of adding dispersion corrections is, however, only minor for **1a**, both qualitatively (Figure 5, left) and quantitatively (Table 2), with overlap estimates 0.68 for B3LYP and 0.63 for B3LYP-D3.

Adding dispersion corrections mainly impacts the relative ordering of the conformers. With dispersion corrections, conformers exhibiting dispersion interactions between two side groups are present: the methyl groups of both alanine residues in **1a** (Figure 6, left) and the Boc and OMe group in **2a** (Figure 6, right). For **1a**, this conformer has a low Boltzmann weight and the spectra with and without dispersion corrections are therefore similar and in agreement with experiment. On the other hand, for **2a** this conformer is the dominating conformer when B3LYP-D3 is used and hence the VCD spectrum for B3LYP-D3 differs from the one without dispersion corrections, leading to a VCD spectrum in poorer agreement with the experimental spectrum.

These results are in agreement with the results of Hopmann et al., who concluded that the Boltzmann distribution based on geometries optimized with dispersion corrections changed the VCD spectrum of a highly flexible natural compound significantly, resulting in poor agreement with experiment.^104^ In Hopmann’s work, enthalpies and free energies result in similar spectra and only spectra with enthalpies were shown. For molecule **2a**, using enthalpies and free energies results in similar spectra while for **1a**, including dispersion corrections and averaging over enthalpies results in a VCD spectrum in poorer agreement with experiment. Koenis et al. also observed that many key VCD bands of a rotaxane had opposite sign compared to the experimental data when using dispersion corrections. Without dispersion corrections, a different conformer of rotaxane dominated and gave a VCD spectrum in good agreement with experiment.^105^ Also Merten and co-workers^106−109^ and Zehnacker and co-workers^110,111^ have shown in several works that including dispersion correction results in significant shifts in the conformational preferences, and that the experimental data are in better agreement with spectra calculated without dispersion corrections. On the basis of these previous observations and our results, it appears that VCD is a sensitive probe of molecular conformation and could hence be used to refine the description of dispersion interactions in quantum-chemical calculations.

### Selection of Significant Conformers

**Figure 7:**
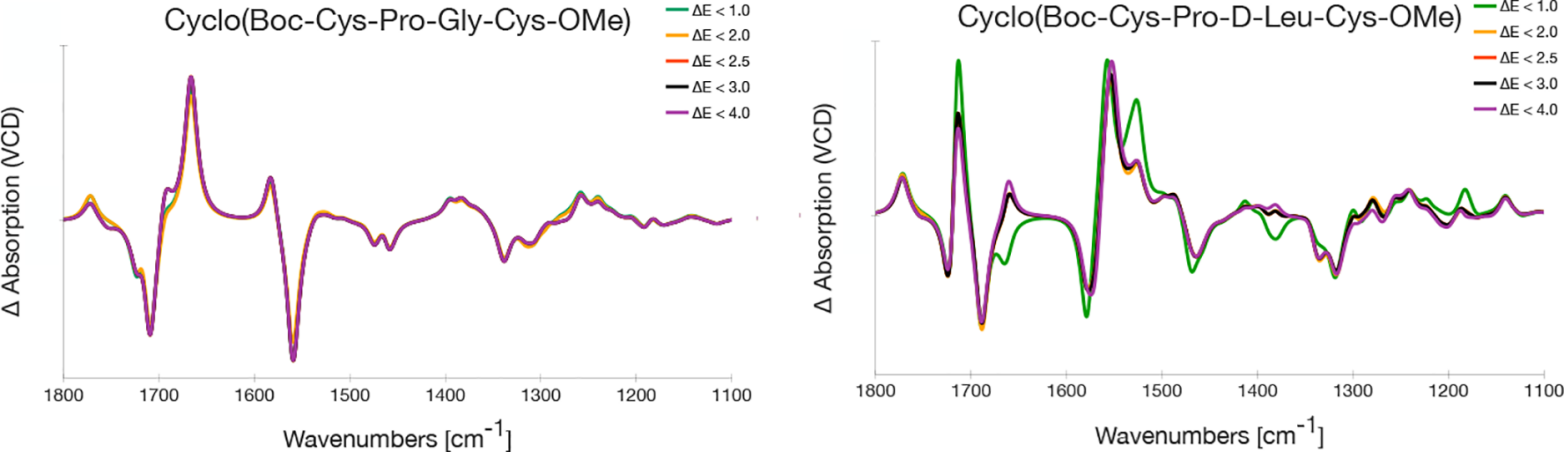
Calculated VCD spectra of **2a** (left) and **2c** (right) in ACN-*d*_3_ based on a different number of conformers. The spectra are based on the conformers within a given energy gap after the single point energy calculations, Δ*E* in kcal/mol, indicated in the figure. The frequencies are not scaled.

To explore the dependence of the calculated VCD spectra on the number of conformers included in the spectral simulations, we consider molecules **2a** and **2c** as these display the largest number of low-energy conformers of the investigated molecules, see Table 3. The results are shown in Figure 7 and Table 4. The number of conformers is based on the difference in energy from the most stable conformer after single-point energy calculations, Δ*E*. Energy gaps of 2.0, 2.5, 3.0, and 4.0 kcal/mol result in qualitatively similar spectra with only small differences in VCD intensities for both molecules (Figure 7). Energy gaps of 2.5 and 3.0 kcal/mol result in an identical number of significant conformers and thus identical VCD spectra. High overlap estimates (*S* ≥ 0.99, Table 4) confirm the high similarity of the spectra.

**Table 3:** Number of Conformers^a^

	conformational sampling	energy ranking		spectra generation	
		single point energy	geometry optimization	BW > 5%	
	CREST	Δ*E* < 2.5 kcal/mol	unique conformers	Δ*H*	Δ*G*
**1a**	7	3	1	1	1
**1b**	73^b^	3	2	2	2
**1c**	56	5	5	3	2
**2a**	331	55	22	10	7
**2b**	669	19	12	5	4
**2c**	778	25	15	9	7
**3**	725	20	20	4	3

a Number of conformers found by CREST, the number of conformers found within 2.5 kcal/mol of the lowest lying conformer after the single point (SP) calculations, the number of unique conformers after DFT geometry optimization and the number of conformers included in the final spectra with a Boltzmann weight (BW) higher than 5%.

b Manually set simulation time, see Table 1.

**Table 4:** Number of Conformers of **2a** and **2c** that Are Geometry Optimized Based on the Energy Gap after the Single Point (SP) Energy Calculation with DFT of the 331 (**2a**) and 778 (**2c**) Conformers Found with CREST^a^

	energy gap (SP) Δ*E* in kcal/mol	conformers geom. opt.	unique conformers	conformers BW > 5%	lowest BW	*S*
**2a**	1.0	9	5	5	5.3%	0.99
	2.0	32	14	6	5.0%	0.99
	2.5	55	22	7	5.0%	1.00
	3.0	68	26	7	5.0%	1.00
	4.0	97	37	7	5.0%	1.00
						
**2c**	1.0	4	3	3	10.3%	0.87
	2.0	13	8	6	5.8%	0.99
	2.5	25	15	7	5.4%	0.99
	3.0	33	16	7	5.4%	0.99
	4.0	66	35	7	5.7%	1.00

a Only conformers with a Boltzmann weight (BW) higher than 5% are included in the spectra. The overlap estimates *S* are calculated with Δ*E* < 4.00 kcal/mol as the reference and over the frequency range shown in Figure 7: 1800–1100 cm^–1^. The frequencies are not scaled.

The number of conformers included in the geometry optimization (Table 4) and thus the main computational cost, depends strongly on the energy threshold. Increasing the energy gap from 2.0 to 2.5 kcal/mol results in one more relevant conformer for both molecules and an overlap estimate of *S* = 1.00 and *S* = 0.99 for **2a** and **2c**, respectively, using the results obtained with an energy threshold of 4.0 kcal/mol as the reference. Increasing the threshold further to 3.0 and 4.0 kcal/mol does not increase the number of relevant conformers, whereas the number of conformers in the geometry optimization increases from 55 to 68 and 97 and from 25 to 33 and 66 for **2a** and **2c**, respectively. An energy gap of 2.5 kcal/mol is also used by Grimme and co-workers in their refining approaches when selecting which conformers to optimize at the DFT level.^80^

### Conformational Analysis

The conformers found by CREST and determined unique and relevant after DFT calculations are in agreement with those found with classical approaches by Vass et al. (**1a**–**c**),^77^ Merten et al. (**2a**–**c**)^78^ and Bour et al. (**3**).^79^**1a**, **1b**, and **1c** adopts for both chiral elements an inverse γ-turn which is in agreement with the findings of Vass et al. For molecule **2a**, we found that the dominating conformers adopts a β_II_ turn structure with small contributions from structures adopting β_*I*_ turns and classical γ turns, whereas Merten et al. assumes that there are significant contributions from both β_I_ and β_II_ type structures. For **2b**, we find all relevant conformers to adopt a β_*I*_ turn structure while **2c** adopts a β_II_ turn structure. These two findings are in agreement with those of Merten et al. The dominating conformers of **3** has two internal hydrogen bonds in the backbone and adopts two β_II_ turns. Also Bour et al. finds that β_II_ types structures make a major contribution to the conformer mix. The computational protocol used in this work confirms findings in the previous studies. Thus, the protocol is able to determine the important conformers of the studied compounds.

### Comparison of Calculated and Experimental Spectra

**Figure 8:**
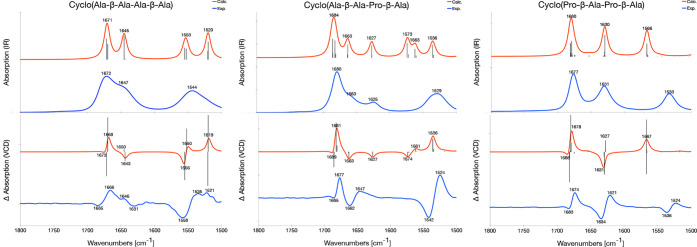
Experimental (blue) and calculated (B3LYP/6-31+G*/CPCM, red) IR (top) and VCD (bottom) spectra of **1a** (left), **1b** (middle), and **1c** (right). For the calculated spectra, a Lorentzian broadening with a full width at half-maximum of 10 cm^–1^ is used and the frequencies for **1a** are scaled with a factor of 0.980 and those for **1b** and **1c** with a factor of 0.990. The experimental spectra are measured by Vass et al.: **1a** in TFE-*d*_2_ while **1b** and **1c** in ACN-*d*_3_.^77^ The sticks in the calculated VCD spectra are the rotational strengths associated with that frequency.

**Figure 9:**
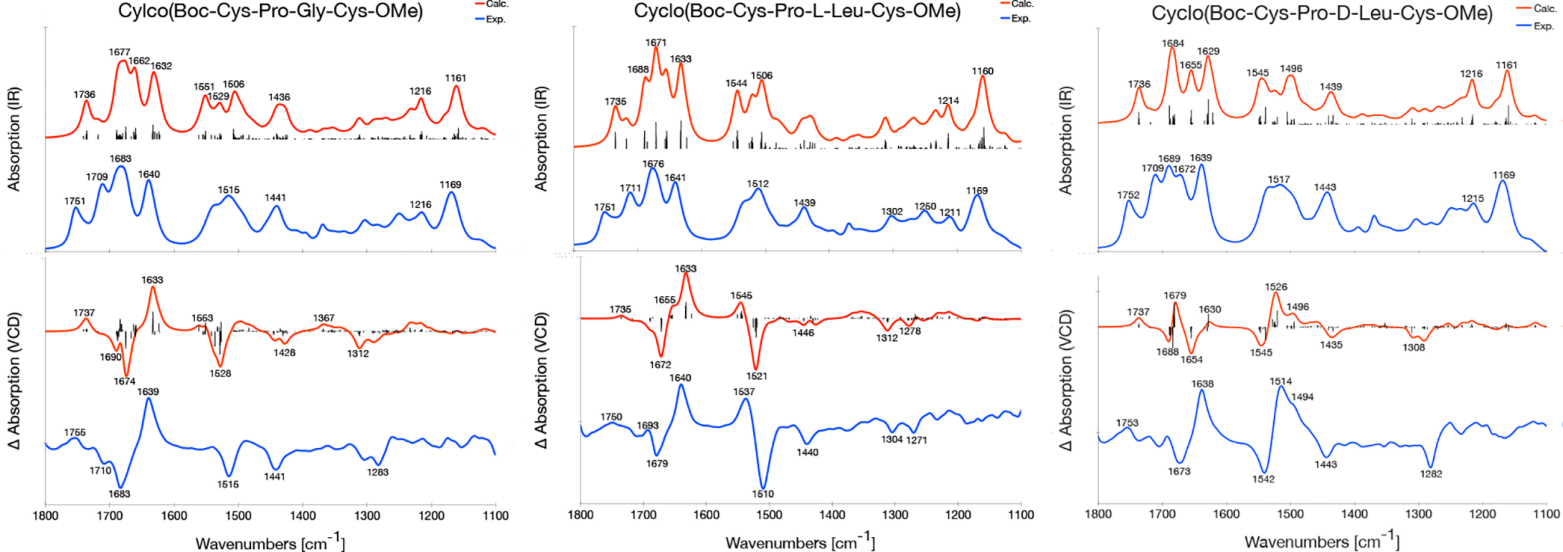
Experimental (blue) and calculated (B3LYP/6-31+G*/CPCM, red) IR (top) and VCD (bottom) spectra of **2a** (left), **2b** (middle) and **2c** (right). For the calculated spectra, a Lorentzian broadening with a full width at half-maximum of 16 cm^–1^ is used, and the frequencies are scaled with a factor of 0.980. The experimental spectra are measured by Merten et al. in ACN-*d*_3_.^78^ The sticks in the calculated VCD spectra are the rotational strengths associated with that frequency.

**Figure 10:**
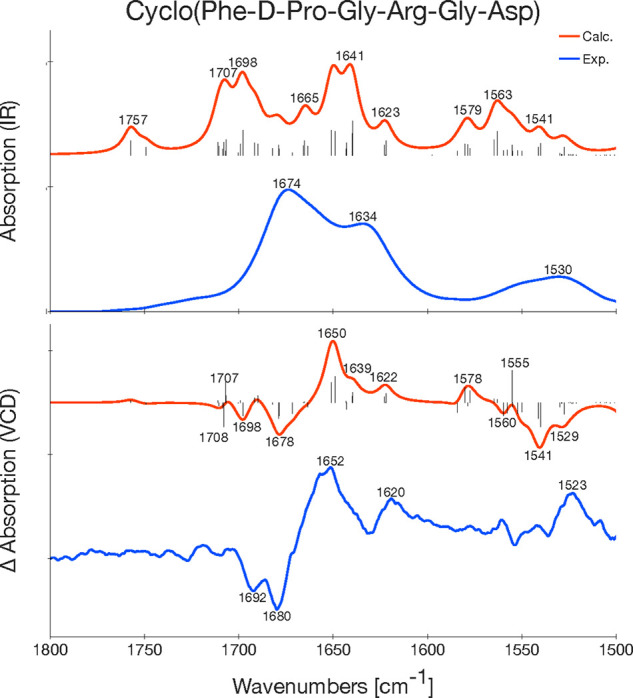
Experimental (blue) and calculated (B3LYP/6-31+G*/CPCM, red) IR (top) and VCD (bottom) spectra of **3**. For the calculated spectrum, a Lorentzian broadening with a full width at half-maximum of 10 cm^–1^ is used and the calculated frequencies are scaled with a factor of 0.995. The experimental spectrum is measured by Bour et al. in TFE.^79^ The sticks in the calculated VCD spectra are the rotational strengths associated with that frequency.

We will now proceed to predict IR and VCD spectra for our selected molecules and compare to experimental spectra. For this, we use the optimized protocol: B3LYP/6-31+G*/CPCM, no dispersion corrections, and a threshold of 2.5 kcal/mol to select the most important conformers after the DFT single-point calculations. The experimental and calculated IR and VCD spectra of **1a**–**c**, **2a**–**c**, and **3** are shown in Figures 8, 9 and 10, respectively, and the overlap estimates *S* are given in Table 5.

**Table 5:** Overlap Estimates *S* between the Calculated (B3LYP/6-31+G*/CPCM) and Experimental IR and VCD Spectra and the Scaling Factor *f* Used for the Spectrum^a^

	*S* (IR)	*S* (VCD)	*f*	freq. range (cm^–1^)
**1a**	0.86	0.68	0.980	1800–1500
**1b**	0.83	0.20	0.990	
**1c**	0.78	0.43	0.990	
**2a**	0.93	0.70	0.980	1800–1100
**2b**	0.94	0.61	0.980	
**2c**	0.92	0.44	0.980	
**3**	0.80	0.31	0.995	1800–1500

a The frequency range used for *S* are the same as shown in the spectra (Figures 8, 9, and 10).

In peptides, there are three important infrared regions. The amide I region from 1800 to 1600 cm^–1^ is characterized by C=O stretching modes, the amide II region from 1600 to 1480 cm^–1^ is characterized by N–H bending modes, and the amide III region from 1350 to 1250 cm^–1^ is characterized by more complex backbone vibrations in combination with side chain vibrations. For **1a**–**c**^77^ and **3**,^79^ only the amide I and II regions have been measured experimentally (1800–1500 cm^–1^) whereas all three regions have been measured (1800–1100 cm^–1^) for **2a**–**c**.^78^

### Molecule Class 1: Cyclo(X-β-Ala-Y-β-Ala)

The shape of the experimental IR spectra of **1a**–**c** are well reproduced by the calculated spectra (Figure 8, top). Considering the VCD spectra of molecule **1a**, both the calculated shape and frequencies of the spectrum are in good agreement with experiment (Figure 8, bottom left panel). The VCD spectrum has a −/+/+/– sign pattern (starting from higher frequencies) in the amide I region which is reproduced well by the calculated spectra. The weak shoulder at 1646 cm^–1^ found in the experiment is in the calculated spectrum only seen when considering the individual rotational strengths (sticks in Figure 8) of the mode at 1650 cm^–1^. The amide II region in the experimental spectrum is rather noisy due to poor transmission of the solvent,^77^ but a −/+ pattern can be seen, which is reproduced in the calculated spectrum. The assignment of the different bands in the IR and VCD spectra of **1a** can be found in Table 6, together with those of **1b** and **1c**.

**Table 6:** Assigned Frequencies (in cm^–1^) of **1a**–**c** from Figure 8^a^

	**1a**		**1b**		**1c**	
	exp	calc	exp	calc	exp	calc
Amide I: C=O stretch						
freq 1	1685 (−)	1673 (−)	1685 (−)	1689 (−)	1683 (−)	1685 (−)
group	Ala, Ala (s)		Ala		Pro, Pro (s)	
freq 2	1666 (+)	1668 (+)	1677 (+)	1681 (+)	1674 (+)	1678 (+)
group	Ala, Ala (as)		Pro		Pro, Pro (as)	
freq 3	1646 (+)	1650 (+)	1662 (−)	1663 (−)	1634 (−)	1631 (−)
group	β-Ala, β-Ala (s)		β-Ala4		β-Ala, β-Ala (s)	
freq 4	1631 (−)	1643 (−)	1647 (+)^b^	1627 (−)	1621 (+)	1627 (+)
group	β-Ala, β-Ala (as)		β-Ala2		β-Ala, β-Ala (as)	
Amide II: N–H bend						
freq 5	1558 (−)	1556 (−)	1542 (−)	1574 (−)	1536 (−)	1566 (−)
group	β-Ala, β-Ala		β-Ala4		β-Ala, β-Ala	
freq 6	1532 (+)	1550 (+)	1524 (+)	1561 (+)	1524 (+)	1566 (+)
group	β-Ala, β-Ala		β-Ala2		β-Ala, β-Ala	
freq 7	–	1520 (−)	–	1536 (+)		
group	Ala, Ala		Ala		–	
freq 8	1521 (+)	1519 (+)				
group	Ala, Ala		–		–	

a All vibrations are assigned to the dominating functional group. Coupled vibrations are indicated as symmetric (s) or asymmetric (as). The sign of the VCD intensities is indicated with (+) or (−). The calculated frequencies for **1a** are scaled with a factor of 0.980 and those for **1b** and **1c** with a factor of 0.990 as in Figure 8.

b Overlap of VCD bands of opposite sign gives uncertain position.^77^

In consideration of **1b**, the signs of the peaks in the VCD experiment are partly reproduced by the calculated spectra. The amide I region in the experimental spectrum has a −/+/–/+ pattern, which is only partly reproduced by the calculated spectrum which displays a −/+/–/– pattern. The experimental IR spectrum has a band at 1625 cm^–1^ which corresponds to the band at 1627 cm^–1^ in the calculated spectrum. In the calculated VCD spectrum, this band is negative, whereas only a broad, positive band at higher frequencies is observed in experiment. In the amide II region, the experimental VCD spectrum only displays two intense bands with a −/+ pattern. In contrast, the calculated spectrum has three separate bands displaying a −/+/+ pattern and with lower intensity.

Turning to **1c**, the experiment is reproduced by the calculations when the individual rotational strengths are considered. The amide I region of the experimental VCD spectrum has a −/+/–/+ pattern which is nicely reproduced by the calculated spectrum. Because the frequencies have been calculated to have too small separation, the band in the calculated spectrum at 1631 cm^–1^ results from two overlapping modes that add up to a single negative band. However, considering the two modes separately, as shown by the sticks at 1631 and 1627 cm^–1^ (Figure 8, lower row panels), the experimental pattern is reproduced by the calculations. In the amide II region of the experimental spectrum there is a −/+ pattern. In the calculated spectrum there is a positive band (1567 cm^–1^) which is in line with the observation from the IR spectra. Again, the frequencies of the two bands have been calculated to have too small separation and the corresponding band in the calculated spectrum at 1567 cm^–1^ consists of two overlapping rotational strengths with different signs, resulting in one positive band in the calculated spectrum (Figure 8, sticks lower right panels).

Although the overall shape of the calculated VCD spectra for **1b** and **1c** is in good agreement with experiment, the overlap estimates are low. Indeed, the overlap estimates for **1b** and **1c** are 0.20 and 0.43, respectively, which is lower than that for **1a** (*S* = 0.68, Table 5). The overlap estimates are very sensitive to small changes in frequencies. For **1b** and **1c**, the calculated gaps between the amide I and amide II regions are much smaller than in the experiments, off by 42 and 34 cm^–1^, respectively. The underestimation of this gap is however only 10 cm^–1^ for **1a**. This results in a low *S* value, particularly for **1b** and **1c**. Using a larger basis set such as 6-311++G** gives amide I–II gaps in slightly better agreement with experiment (top row Figure S1 and Figure S3). In addition, earlier work has suggested that including explicit solute–solvent interactions results in an amide I–II gap in better agreement with experiment.^79,112^

For **1b** and **1c**, we also note that some of the calculated modes are too close in frequency, which merges these vibrational modes into a single, broader band shape, and the VCD stick spectrum is needed to allow for a more detailed comparison with experiment (Figure 8, bottom middle and bottom right panels).

### Molecule Class 2: Cyclo(Boc-Cys-Pro-X-Cys-OMe)

The experimental IR and VCD spectra of **2a**–**c** are reproduced by the calculated spectra (Figure 9). Starting with **2a**, the amide I region of the experimental VCD spectrum (Figure 9, bottom left) has a +/–/+ sign pattern, which is reproduced by the calculations. In the amide II region, the experiment has a negative band at 1515 cm^–1^, which corresponds to the calculated peak at 1528 cm^–1^. Also, at lower frequencies the calculations are in good agreement with experiment. The assignment of the different bands in the IR and VCD spectra of **2a** can be found in Table 7, together with those of **2b** and **2c**.

**Table 7:** Assigned Frequencies (in cm^–1^) of **2a**–**c** from Figure 9^a^

	**2a**		**2b**		**2c**	
	exp	calc	exp	calc	exp	calc
Amide I: C=O stetch						
freq 1	1755 (+)	1737 (+)	1750 (+)	1735 (+)	1755 (+)	1737 (+)
group	Cys4		Cys4		Cys4	
freq 2	1710 (−)	1690 (−)	1711 (−)	1689 (−)	–	1688 (−)
group	Boc		Boc		Boc	
freq 3	1683 (−)	1674 (−)	1679 (−)	1672 (−)	–	1679 (+)
group	Pro		Pro		Pro	
freq 4	–	1660 (+)	–	1655 (+)	1673 (−)	1654 (−)
group	Gly		l-Leu		d-Leu	
freq 5	1639 (+)	1633 (+)	1640 (+)	1633 (+)	1638 (+)	1630 (+)
group	Cys1		Cys1		Cys1	
Amide II: N–H bend						
freq 6	1541 (+)	1553 (+)	1537 (+)	1545 (+)	1542 (−)	1545 (−)
group	Cys4		Cys4		Cys4	
freq 7	1515 (−)	1528 (−)	1510 (−)	1521 (−)	1513 (+)	1526 (+)
group	Gly		l-Leu		d-Leu	
freq 8	1487 (+)	1498 (+)	–	1483 (+)	1494 (+)^b^	1496 (+)
group	Cys1		Cys1		Cys1	

a All vibrations are assigned to the dominating functional group. The sign of the VCD intensities is indicated with (+) or (−). The calculated frequencies are scaled with a factor of 0.980 as in Figure 9.

b Shoulder.

For **2b**, both the experimental and the calculated VCD spectrum have a −/+ pattern in the amide I region and a +/– pattern in the amide II region (Figure 9, bottom middle). Also, at lower frequencies the calculated spectrum is in agreement with the experimental spectrum.

For **2c**, the sign of the bands in the experimental VCD spectrum are reproduced by the calculations when the individual sticks are considered (Figure 9, bottom right). In the experimental VCD spectrum, there are two main peaks in the amide I region: negative at 1673 cm^–1^ and positive at 1638 cm^–1^. The negative peak is reproduced in the calculated spectrum at 1654 cm^–1^ whereas the positive peak appears as a stick at 1630 cm^–1^. In the calculated spectrum, there is a strong positive band at 1679 cm^–1^ that is a much weaker shoulder at 1692 cm^–1^ in the experiment. The −/+ sign pattern of the amide II region is well reproduced in the calculated spectrum.

The overlap estimates of the VCD spectra for **2a**, **2b**, and **2c** are 0.70, 0.61, and 0.44, respectively. As for **1a**–**c**, the gap between the amide I and amide II region is underestimated by approximately 20 cm^–1^ with respect to the experimental spectrum. Although the shape of the amide I region for **2c** is in agreement with experiment, the intensities are not and this, combined with the too small amide I–II gap, leads to the significantly lower overlap estimate for **2c**.

### Molecule 3: Cyclo(Phe-d-Pro-Gly-Arg-Gly-Asp)

Molecule **3** (Figure 3) is a hexapeptide with four chiral centers and more conformational flexibility in the side chains compared to molecules **1**–**2**. The experimental IR and VCD spectra of **3** are reproduced quite well by the calculations, though there are only a few distinct bands in the experimental IR spectrum, making a detailed comparison difficult. The amide I region of the of the experimental VCD spectrum (Figure 10) has a – /–/+/+ pattern, which is is also found in the calculated spectrum. The negative peaks can be assigned to a C=O stretch on Arg and Asp, respectively, whereas the positive peaks can be assigned to a C=O stretch on Phe (strong positive peak at 1652 cm^–1^) and different stretches and bends in the side groups of Phe and Arg (positive peak at 1620 cm^–1^), see Table 8.

**Table 8:** Assigned Frequencies (in cm^–1^) of **3** from Figure 10^a^

	**3**	
	exp	calc
Amide I: C=O stretch		
freq 1	–	1711 (−)
group	Pro	
freq 2	–	1708 (−), 1707 (+)^b^^,^^c^
group	Gly5	
freq 3	1692 (−)	1698 (−)
group	Arg	
freq 4	1680 (−)	1678 (−)
group	Asp	
freq 5	1652 (+)	1650 (+)
group	Phe	
freq 6	–	1639 (+)^c^^,^^d^
group	Gly3	
freq 7	1620 (+)	1622 (+)
group	side chains on Phe and Arg	

a All vibrations are assigned to the dominating functional group. The sign of the VCD intensities is indicated with (+) or (−). The calculated frequencies are scaled with a factor of 0.995 as in Figure 10.

b Two modes in combination with N–H bend on side group with different sign of the VCD intensity.

c Different conformers give opposite sign for the VCD intensity.

d Shoulder.

In the amide II region, there is one strong band at 1523 cm^–1^ in the experimental VCD spectrum. This peak is reproduced by a strong absorption at 1555 cm^–1^ in the calculated VCD stick spectrum. The frequency shift of this peak is one reason for the rather low overlap between calculated and experimental spectra both for IR (*S* = 0.80) and VCD (*S* = 0.31), see Table 5.

## Summary and Outlook

We have investigated the performance of the conformational search tool CREST developed by Grimme and co-workers,^69^ originally developed for quantum chemical calculation of NMR spectra, for the calculation of IR and VCD spectra of seven conformationally flexible cyclic oligopeptides for which experimental spectra are available. Chiroptical properties of flexible molecules are particularly challenging, as different conformers may have different signs of the rotatory strengths, in contrast to, for instance, IR or NMR intensities. Ensuring that all relevant conformers are identified and their relative stability is correctly predicted is therefore paramount for a robust computational protocol to reproduce experimental VCD spectra.

Our results show that CREST identifies the important conformers. Subsequent DFT energy calculations ensure sufficiently accurate results for the relative energies of the different conformers and lead to a reduction of the number of conformers for which a full geometry optimization and VCD properties calculations need to be performed. In weighing the overall contribution of different conformers to generate full IR and VCD spectra, Gibb’s free energies are used. We note, however, that in many cases the enthalpy will suffice if the different conformers do not display significantly different entropic contributions. Somewhat surprisingly, but confirming earlier VCD studies,^104,105^ including dispersion corrections leads to poorer agreement with experimental VCD spectra. This suggests that the sensitivity of VCD may make it an appropriate technique to use when attempting to further improve the description of dispersion corrections in DFT calculations.

The overall VCD patterns computed based on the conformers identified by the approach are in good agreement with available experimental data. However, the gap between the amide I and amide II region does not match experiment for the molecules investigated in this work. Whereas we have used continuum solvation models, explicit solvation may be required in order to correctly model the energy separation between the amide I and amide II regions.^79^ Furthermore, some vibrational bands are too close in frequency in our calculations, leading to overlapping vibrational bands in the calculated VCD spectra that may hide finer patterns observed in the experimental spectra. However, these patterns can be identified by considering the computed spectrum without broadening applied (the stick spectrum). Anharmonic corrections may also also be relevant to consider in some cases.^113,114^

Despite these shortcomings, the tested protocol is able to identify the relevant conformers and the calculated VCD spectra are overall in agreement with experiments. The approach provides a feasible basis for generating VCD spectra also for larger cyclic peptides of biological/pharmaceutical interest. Whereas our protocol has been able to reproduce experimental VCD spectra of these cyclic peptides with known stereochemistry, an interesting question is to what extent experimental VCD in combination with the current computational protocol will be able to distinguish structurally similar cyclic peptides that have different stereochemistry. This will be the focus of future work.

## Supporting Information Available

Basis set test for cyclic oligopeptides; test of different scaling factors; comparison of using enthalpies and free energies in the Boltzmann averaged spectra (PDF)
