# Supplementary material for: A Computational Protocol for Vibrational Circular Dichroism Spectra of Cyclic Oligopeptides
Source: J Phys Chem A. 2022 Aug 5;126(32):5458–71. doi: 10.1021/acs.jpca.2c02953 (PMC9393892; doi:10.1021/acs.jpca.2c02953)
Supplement: Supplementary file 1 — jp2c02953_si_001.pdf [file jp2c02953_si_001.pdf]

Supporting Information: Supporting Information for "A Computational Protocol for VCD spectra of Cyclic Oligopeptides"

Karolina Di Remigio Eikås,[*,†] Maarten T. P. Beerepoot,[†] and Kenneth Ruud[*,†,‡]

†*Hylleraas Centre for Quantum Molecular Sciences, Department of Chemistry, UiT The Arctic University of Norway, 9037 Tromsø, Norway*

‡*Norwegian Defence Research Establishment, P.O. Box 25, 2027 Kjeller, Norway*

E-mail: karolina.s.eikas@uit.no; kenneth.ruud@uit.no

Basis set dependence

VCD spectra and overlap estimates S for **1a** and **2a** calculated with different basis sets are shown in Figure S1 and Table S1, respectively. The two molecules show the same trends with respect to the effects of contraction scheme, polarization functions and diffuse functions. A less extended basis set test was performed for **1b** and **1c**. The trends for these two molecules are the same as for **1a** and **2a** and the results are collected in the SI.

VCD spectra calculated using double-zeta and triple-zeta basis sets are very similar (top row in Figure S1). The addition of a first set of polarization and diffuse functions on the heavy atoms impacts the VCD spectra significantly, whereas adding diffuse and polarization functions also on hydrogen atoms has only a minor effect. Polarization functions are the most important as the spectra calculated for both molecules without polarization functions

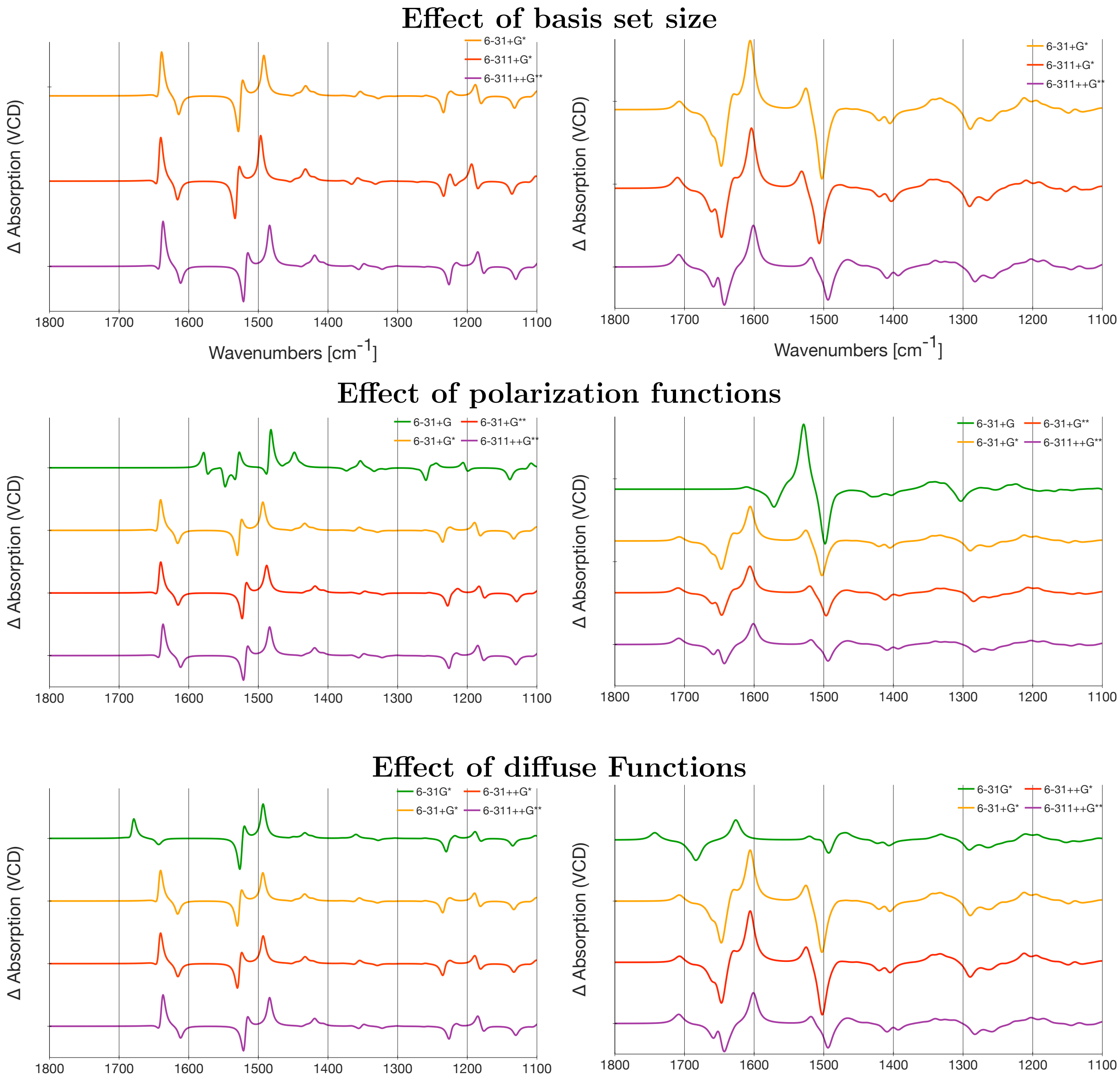

Figure S1: Calculated VCD spectra for molecules **1a** (left) and **2a** (right). The calculations are performed with B3LYP/CPCM and different basis sets. The frequencies were scaled with a factor of 0.9613 for 6-31G*, 0.9636 for 6-31+G, 6-31+G* and 6-31++G*, 0.9648 for 6-31+G**, 0.9673 for 6-311++G**, and 0.9680 for 6-311+G*.[S1]

Table S1: Overlap estimate S between calculated IR and VCD spectra for molecules **1a** (left) and **2a** (right). The calculations are performed with B3LYP/CPCM and different basis sets. S (Eq. 1) is calculated over the frequency range 1800-1500 cm^{-1} for **1a** and 1800-1100 cm^{-1} for **2a**, using 6-311++G** as the reference. The frequencies were scaled with a factor of 0.9613 for 6-31G*, 0.9636 for 6-31+G, 6-31+G* and 6-31++G*, 0.9648 for 6-31+G**, 0.9673 for 6-311++G**, and 0.9680 for 6-311+G*.[S1]

	Molecule **1a**			Molecule **2a**		
Basis set	Basis functions	IR	VCD	Basis functions	IR	VCD
Effect of basis set size						
6-31+G*	420	0.80	0.44	676	0.96	0.80
6-311+G*	500	0.74	0.37	810	0.97	0.75
6-311++G**	580	1.00	1.00	930	1.00	1.00
Effect of polarization functions						
6-31+G	340	0.44	0.26	548	0.57	0.30
6-31+G*	420	0.80	0.44	676	0.96	0.80
6-31+G**	440	0.92	0.82	706	0.98	0.91
6-311++G**	580	1.00	1.00	930	1.00	1.00
Effect of diffuse functions						
6-31G*	300	0.53	0.33	484	0.75	0.30
6-31+G*	420	0.80	0.44	676	0.96	0.80
6-31++G*	480	0.80	0.45	766	0.96	0.80
6-311++G**	580	1.00	1.00	930	1.00	1.00

(6-31+G) are qualitatively different to all other calculated spectra (Figure S1, top). This observation is confirmed by the overlap estimates in Table S1, which increase from 0.26 to 0.44 for **1a** and from 0.30 to 0.80 for **2a** upon the addition of the first set of polarization functions. The overlap estimates using 6-31+G*, 6-31++G* and 6-311+G* for **1a** are rather low for both the IR and VCD spectra compared to **2a**. This is due to changes in the frequency gap between the amide I and amide II regions. In fact, the frequencies in the amide I region for the three basis sets agree well with the spectrum calculated with 6-311++G**, whereas there is a small shift towards lower frequencies in the range 1600-1100 cm^{-1} (Figure S3).

Bour *et al.* found that the VCD band shape of a cyclic hexapeptide was in general very similar using 6-311++G** and 6-31G**, but that 6-311++G** provided slightly lower frequencies. This was most pronounced in the amide I region, and the addition of diffuse functions led to better agreement with experiment.[S2] The latter observation is in agreement with our results (bottom row Figure S1). Indeed, especially the first set of diffuse functions leads to a decrease of the frequency gap between the amide I and amide II regions and hence to a better agreement with experiment.

Changing the basis set from 6-31+G* to 6-311++G** for **1a** and **2a**, however, leads to an increase in the number of basis functions from 420 to 580 and 676 to 930, respectively, for **1a** and **2a**. Since 6-31+G* gives qualitatively similar spectra to 6-31++G*, 6-31+G** and 6-311+G*, we conclude that this basis set is a good compromise between accuracy and computational efficiency. This is particularly important as our longer-term goal is to investigate much larger peptides. For this reason, the 6-31+G* basis set will be used in the remainder of this paper.

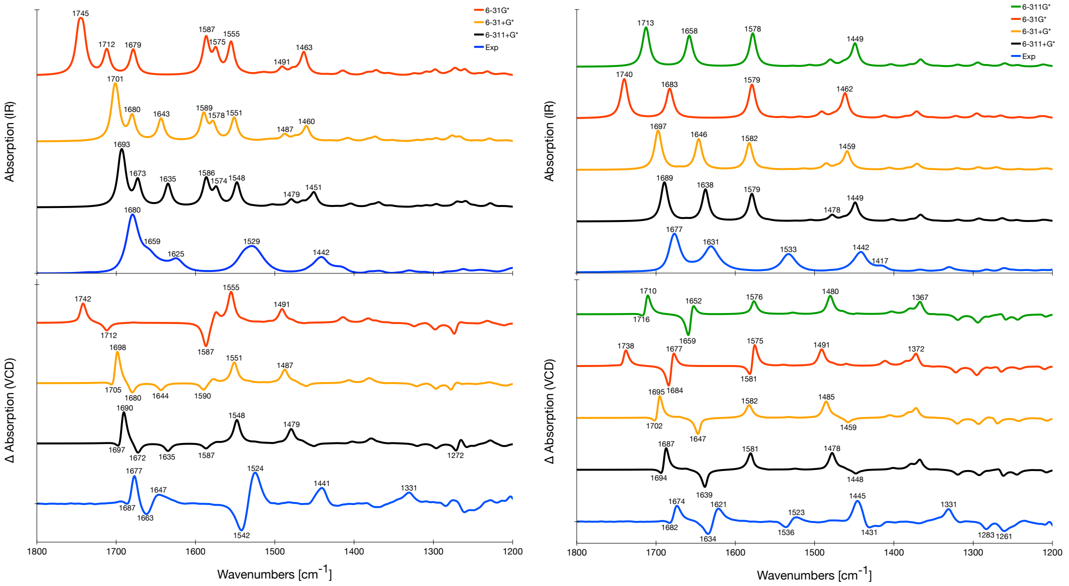

Figure S2: Experimental and calculated IR and VCD spectra of **1b** (left) and **1c** (right). The calculations are done with B3LYP, the basis set indicated in the figure and CPCM. For the calculated spectra a Lorentzian broadening with a full-width at half-maximum of 10 cm^{-1} is used and the frequencies are not scaled. The experimental spectra are measured by Vass *et al.* in ACN-d_3.[S3]

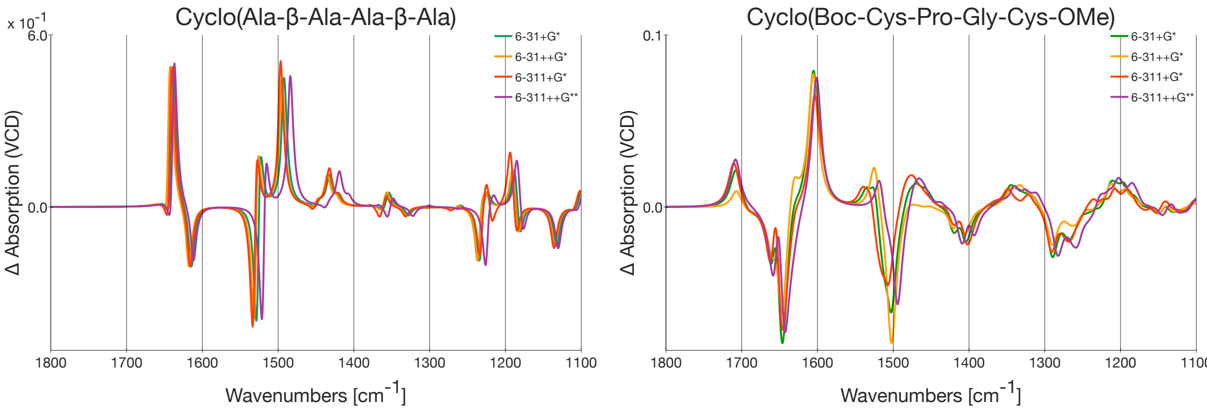

Figure S3: Calculated VCD spectra of **1a** and **2a** with B3LYP and the basis set indicated in the Figure. For **1a** the solvent model CPCM = TFE is used while for **2a** CPCM=ACN is used. A Lorentzian broadening with a full-width at half-maximum of 10 cm^{-1} for **1a** and 16 cm^{-1} for **2a** is used. The frequencies are scaled with 0.9636 for 6-31+G* and 6-31++G*, 0.9680 for 6-311+G* and 0.9673 for 6-311++G**.[S1]

Scaling factor

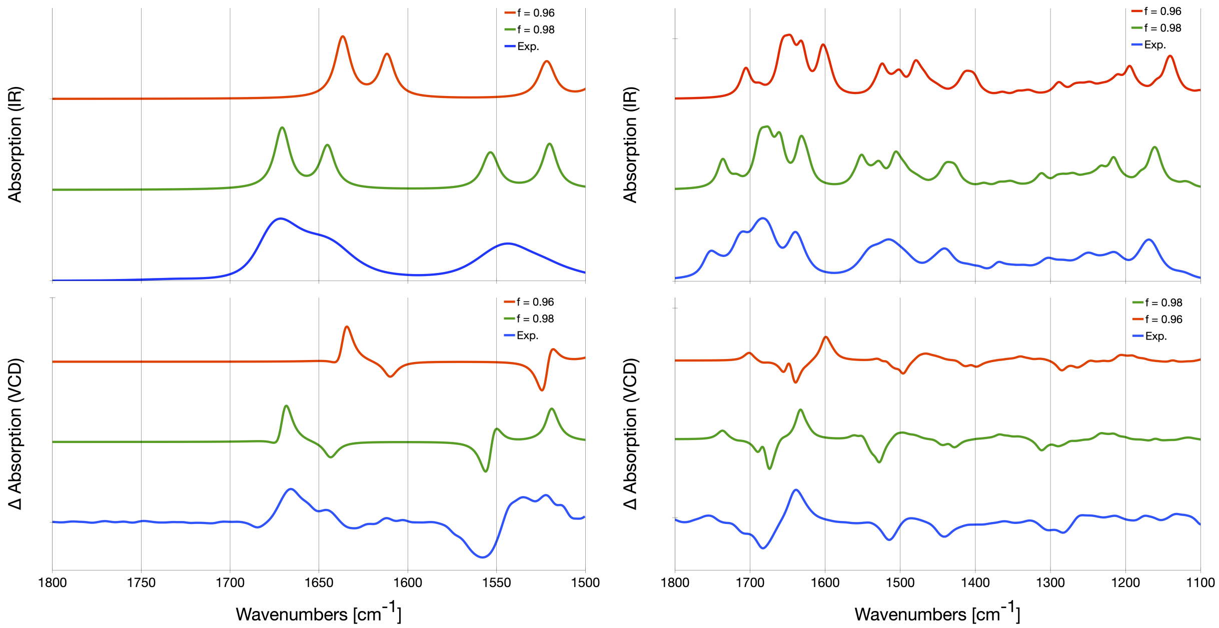

Figure S4: IR and VCD spectra of molecules **1a** (left) and **2a** (right) with different scaling factor f for the calculated (B3LYP/6-31+G*/CPCM) spectra, compared with experiment.

Table S2: Overlap estimate S between calculated and experimental IR and VCD spectra using different scaling factor f. S is calculated over the frequency range shown i Figure S4: 1800-1500 cm^{-1} for **1a** and 1800-1100 cm^{-1} for **2a**.

	Molecule **1a**		Molecule **2a**	
f	IR	VCD	IR	VCD
0.96	0.48	-0.19	0.77	-0.17
0.97	0.69	0.19	0.84	0.36
0.98	0.86	0.68	0.93	0.67
0.99	0.71	0.11	0.95	0.50
1.00	0.51	-0.20	0.87	0.08

The effect of using different frequency scaling factors on the spectra for **1a** and **2a** are shown in Figure S4 (IR and VCD spectra) and Table S2 (overlap estimates S). The two molecules show the same trends when it comes to scaling factor, with the overlap estimate for both the IR and VCD spectra going through a maximum for a scaling factor of 0.98.

Merrick, Moran and Radom recommended a scaling factor for B3LYP/6-31+G* of 0.9636 based on a comparison with experimental gas-phase data.[S1] Our calculations for **1a** and **2a**

have instead been compared to solution spectra using respectively trifluoroethyl alcohol (TFE) and acetonitrile (ACN) as solvents, and shows that scaling the frequencies by 0.96 gives poor agreement with experiment, both quantitatively (Figure S4) and qualitatively (Table S2). Indeed, the negative S-values for the VCD spectra indicate that the calculated VCD spectra have more spectral overlap with the enantiomer of the investigated molecules. A scaling factor of 0.98 gives the best agreement between the calculated and experimental spectra for both molecules (Table S2). Poor agreement between experimental and calculated spectra using gas-phase fitted scaling factors has also been previously noted for solvated systems.[S4–S6] We note, however, that peptides pose a particular challenge when comparing to experiment, as the amide I–II gap cannot be reliably reproduced without taking solute-solvent interactions explicitly into account, and the scaling factor thus becomes a compromise in our description of these important bands.[S2] Whenever comparing to experimental spectra, the frequencies are scaled with the factor giving the largest overlap estimate S between the calculated and experimental spectra.

Enthalpies and Gibbs free energies in the Boltzmann weight

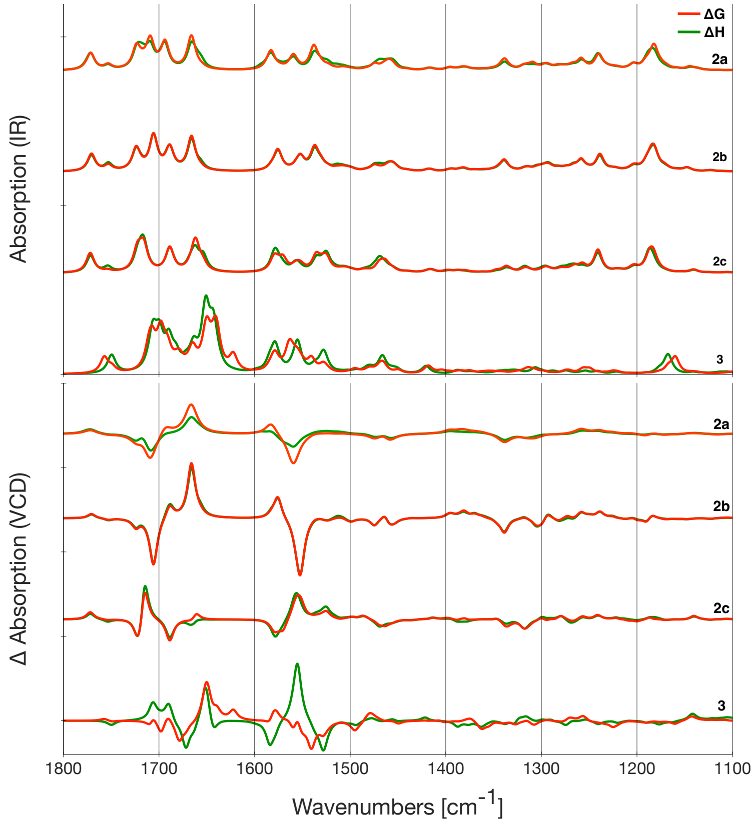

Figure S5: Calculated VCD spectra of **2a**, **2b**, **2c** and **3** with free energies (ΔG, red) and entalpies (ΔH, green) in the Boltzmann weights. The calculations are done with B3LYP, 6-31+G* and CPCM.

Table S3: The overlap estimate S between the calculated and the experimental VCD spectra of **2a-c** and **3** with entalpies and free energies used in the Boltzmann weight.

	**2a**	**2b**	**2c**	**3**
ΔH	0.67	0.61	0.33	0.01
ΔG	0.70	0.61	0.44	0.31

References

(S1) Merrick, J. P.; Moran, D.; Radom, L. An Evaluation of Harmonic Vibrational Frequency Scale Factors. *J. Phys. Chem. A* **2007**, *111*, 11683–11700.

(S2) Bouř, P.; Kim, J.; Kapitan, J.; Hammer, R.; Huang, R.; Wu, L.; Keiderling, T. Vibrational circular dichroism and IR spectral analysis as a test of theoretical conformational modeling for a cyclic hexapeptide. *Chirality* **2008**, *20*, 1104–19.

(S3) Vass, E.; Majer, Z.; Kőhalmy, K.; Hollósi, M. Vibrational and chiroptical spectroscopic characterization of γ-turn model cyclic tetrapeptides containing two β-Ala residues. *Chirality* **2010**, *22*, 762–771.

(S4) Mennucci, B.; Martínez, J. M. How to Model Solvation of Peptides? Insights from a Quantum-mechanical and Molecular Dynamics Study of N-Methylacetamide. 1. Geometries, Infrared, and Ultraviolet Spectra in Water. *J. Phys. Chem. B* **2005**, *109*, 9818–9829.

(S5) Cappelli, C.; Silva, C. O.; Tomasi, J. Solvent effects on vibrational modes: ab-initio calculations, scaling and solvent functions with applications to the carbonyl stretch of dialkyl ketones. *Journal of Molecular Structure: THEOCHEM* **2001**, *544*, 191 – 203.

(S6) Koenis, M. A. J.; Visser, O.; Visscher, L.; Buma, W. J.; Nicu, V. P. GUI Implementation of VCDtools, A Program to Analyze Computed Vibrational Circular Dichroism Spectra. *J. Chem. Inf. Model.* **2020**, *60*, 259–267.
